# Willingness to pay for private health insurance among workers with mandatory social health insurance in Mongolia

**DOI:** 10.1186/s12939-020-01343-9

**Published:** 2021-01-06

**Authors:** Ochirbat Batbold, Christy Pu

**Affiliations:** 1 Ach Medical University, Peace Avenue-11, Songino-Khairkhan district-18, Ulaanbaatar, Mongolia; 2 Institute of Public Health, National Yang-Ming University, (112) 155 Linong St. Sec 2, Peitou, Taipei, Taiwan

**Keywords:** Social health insurance, Private health insurance, Willingness-to-pay, Contingent valuation, Universal coverage

## Abstract

**Background:**

High out-of-pocket health expenditure is a common problem in developing countries. The employed population, rather than the general population, can be considered the main contributor to healthcare financing in many developing countries. We investigated the feasibility of a parallel private health insurance package for the working population in Ulaanbaatar as a means toward universal health coverage in Mongolia.

**Methods:**

This cross-sectional study used a purposive sampling method to collect primary data from workers in public and primary sectors in Ulaanbaatar. Willingness to pay (WTP) was evaluated using a contingent valuation method and a double-bounded dichotomous choice elicitation questionnaire. A final sample of 1657 workers was analyzed. Perceptions of current social health insurance were evaluated. To analyze WTP, we performed a 2-part model and computed the full marginal effects using both intensive and extensive margins. Disparities in WTP stratified by industry and gender were analyzed.

**Results:**

Only < 40% of the participants were satisfied with the current mandatory social health insurance in Mongolia. Low quality of service was a major source of dissatisfaction. The predicted WTP for the parallel private health insurance for men and women was Mongolian Tugrik (₮)16,369 (*p* < 0.001) and ₮16,661 (*p* < 0.001), respectively, accounting for approximately 2.4% of the median or 1.7% of the average salary in the country. The highest predicted WTP was found for workers from the education industry (₮22,675, SE = 3346). Income and past or current medical expenditures were significantly associated with WTP.

**Conclusion:**

To reduce out-of-pocket health expenditure among the working population in Ulaanbaatar, Mongolia, supplementary parallel health insurance is feasible given the predicted WTP. However, given high variations among different industries and sectors, different incentives may be required for participation.

## Background

The achievement of universal health care (UHC) coverage, which is one of United Nations’ Sustainable Development Goals (SDG) [1] to reverse the impoverishing effects of out-of-pocket (OOP) health expenditures, is a challenge faced by many developing countries, including Mongolia.

Mongolia introduced social health insurance (SHI) in 1994 to achieve UHC; it is compulsory for all public and private sector employees and voluntary for unemployed people. The type of contribution is income-based and is equal to 4% of the insurant’s monthly salary, which is equally shared between the employer and employee [2]. The SHI package, however, covers only limited inpatient and outpatient services in public and some contracted private health care providers [3]. The coverage provided by the Mongolian SHI is highly inadequate. In Mongolia, OOP expenditure accounted for 41% of the total health expenditure in 2011, causing a severe household financial burden [2].

Moreover, the Mongolian population relies heavily on private health care providers because of service delivery failures in the public sector, including complicated hospital admission practices, poor referral and appointment system, and long waiting times [4]. This high level of dissatisfaction with public health care services is common in developing countries [5]. Private health care providers are often perceived to offer better services, technology, and ease of access [6, 7]. A high utilization rate of private health care services exists among workers. However, these services are not fully covered by the existing SHI system in Mongolia, which can result in considerable and often unpredictable health care OOP expenditure [2, 8–10].

Studies on the use of private health insurance (PHI) as a means to achieve UHC are limited. Most previous studies have focused on the use of national or public health insurance programs as a means of achieving UHC [11]. However, PHI could play a positive role in improving health financing when it complements the existing SHI, especially when the SHI provides only limited coverage [12, 13].

In this study, we assessed the demand of PHI among workers from different industries in Mongolia by analyzing their willingness to pay (WTP) for PHI. Most of the previous studies investigating WTP for PHI have often focused on the general population. In the case of Mongolia, focusing on employees, instead of the general population, is a more feasible approach for several reasons. First, Mongolia has a very high proportion (65%) of working-age population (i.e., female and male citizens aged 15 to 55 or 60 years, respectively), which can be viewed as an enormous demographic window according to the 2018 National Statistical Office (NSO) Report [14]. Therefore, relying on the employed population to finance health care expenditure is considered more feasible. Notably, the employed population contributes the most to healthcare financing in several countries [13].

Second, Mongolia has successfully transitioned into a market-oriented economy since 1990. During the transition period, the employment rate decreased sharply from 87 to 62% until 2001 [15]. After the transition, the Mongolian economy started heavily exploiting its mineral deposits [16], shifting away from its previous considerable dependence on the agriculture sector, in which the employment rate was 40.3% in 2007 and decreased to 27.8% in 2014 [17]. These shifts exacerbated the existing disparities among workers from all industries. The traditional labor market is concentrated in the mining sector. Therefore, examining the WTP of employees from the different sectors is essential for determining the feasibility of the parallel health insurance system. Without sufficient insights into the WTP of employees from various industries (rather than the general population), establishing a well-functioning UHC/SHI system will be challenging. This may not be the case for countries where the government implements universal health insurance with mandatory enrollment, regardless of employment [18].

Third, Mongolia’s labor market is characterized by high mobility. High mobility and low tenure in the labor market can be addressed by the parallel PHI system, as it can be provided as a job incentive and can effectively attract talent from the labor market [19].

Our study assessed the Mongolian population’s current satisfaction levels with the mandatory SHI and analyzed the WTP for private parallel insurance across industries. This was an exploratory study of the working population in Mongolia. Considering the high gender employment inequality prevalent in Mongolia [15, 20, 21], we also tested any gender gaps in our estimates to highlight the gender disparities among employees in Mongolia.

## Methods

### Participants

We conducted a cross-sectional survey between July and September 2018 in Ulaanbaatar, Mongolia. We used purposive sampling to collect data from 22 public and private companies from 11 industries in Ulaanbaatar: mining, processing, electricity, construction, wholesale and retail, transportation, information and communication, finance and insurance, public administration, education, and health care. A total of 83.3% of all employees in Ulaanbaatar work in these 11 industries, representing both the public and private sectors [22]. We aimed to involve the organizations that represent Mongolian industries based on their market share based on the advice of the Mongolian National Chamber of Commerce and Industry. The content validity of the questionnaire was examined by public health experts, statistical specialists, and professors from National Yang-Ming University (Taiwan) and Ach Medical University (Mongolia). This study was approved by the Institutional Review Board of National Yang-Ming University (YM107064E-2), Taipei, Taiwan, and by the Medical Ethics Committee of Ach Medical University (12/23), Ulaanbaatar, Mongolia.

The working population in Ulaanbaatar in 2018 was estimated by the NSO to be 555,350 people, with 303,544 men and 251,806 women. The sample size *n* for our study was determined using Slovin’s formula for a known population [23, 24]:

$$ n=\frac{N}{1+N{e}^2} $$

where *N* is the population size (555350), and *e* is the level of precision (set as 0.05). Considering the response rate and missing data, we assigned a higher number, and 1925 full-time employees from 11 industries, 18 private companies, and 4 public administrations agreed and accepted our invitation letters to participate in the study and completed the questionnaire (overall response rate = 86.1%). After excluding 268 individuals with missing data, we included 1657 participants.

### Perception of the current social health insurance system

To determine the WTP questions for private health insurance, first, we obtained the participants’ perception of the current mandatory social health insurance system by using the following questions:

1. *Are you satisfied with social health insurance? (Yes/No);*
2. *Please tick one best thing you like about social health insurance (premium/quality/level of convenience/other);*
3. *Please tick one worst thing you dislike about social health insurance (premium/quality/level of convenience/other);*
4. *What would you like to improve in the social health insurance system if you had a chance to do so? (service/quality/health care provider/premium/other)*

### WTP for parallel private health insurance

We used the contingent valuation (CV) method to examine the preferences of individuals to determine WTP [25, 26]. We used a double-bounded dichotomous choice elicitation questionnaire, as used in studies to determine WTP for private health insurance in low- and middle-income countries [25, 27].

### Hypothetical private health insurance package

**Fig. 1:**
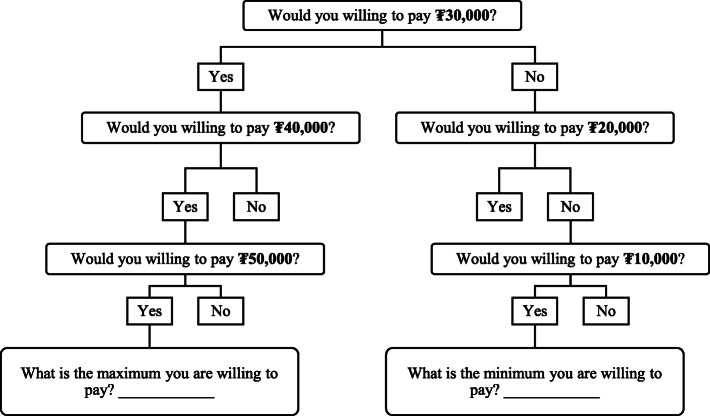
Willingness to pay flow diagram

We proposed the following private health insurance package with total coverage of 5 times higher (Mongolian Tugrik [MNT or ₮] 10,000,000 or US$4167) than the present annual coverage by social health insurance. This amount was set after discussion with public health experts and health economists in Mongolia, who believe such an amount is required for reasonable financial protection. In the proposed package (explained below), the coverage would include the cost of health services and drugs not covered by SHI. We set the starting premium at ₮30,000 to exemplify future similar insurance products. The premium amount was equal to 5% of the median salary (₮670,300 in 2017 according to the NSO), which is a reasonable percentage based on Asian countries that have achieved UHC successfully [28]. The proposed package was laid out in the following manner:

*How much would you pay for private medical insurance if such a product were available? Here, we assume that such private medical insurance will reimburse you up to ₮10,000,000 for hospitalization and other medical costs (*e.g.*, outpatient, inpatient, drug costs, laboratory test) not covered by the current social health insurance. This amount can be used on yourself only and not transferable to your family members, who will each have to use a separate policy. Please look at the price of the premium below and tell us whether you will be willing to pay that amount of premium per month* (Fig. 1).

### Feasibility assessment

Our proposed package is aimed to be provided by private health insurance companies. Thus, the product must be profitable so that private companies would be willing to offer the product. After consultations with private insurance companies’ health insurance specialists, the premium and benefit package we offered was more competitive than the existing private insurance products in Mongolia. The offered package was thus deemed profitable after actuarial calculation.

Studies using a CV survey indicated that WTP that the respondents state on behalf of their households is not significantly different from their individual WTP [29]. However, it may vary depending on household and respondent’s characteristics, but the main factor is a clear description of the good in the WTP question [30]. The current social health insurance premium is set on an individual basis and not on a household basis, as in other countries [29, 31, 32]. We specifically made the package nontransferable to family members, as the objective of this package is to improve the health of the working population instead of the general population.

Similar to other studies on WTP for developing countries [25, 26], we set the initial premium as ₮30,000 (1 USD ≈ ₮2400 in 2018, which means approximately US$12 per month) and then subtracted and added ₮10,000 to the initial bid to estimate WTP.

### Other covariates

We chose relevant covariates by using a model-building process. First, we selected relevant covariates from the literature and retained those with *P* < .2 as potential candidate covariates. As we wished to test gender differences, each covariate was interacted with the gender variable. We then discarded variables that were not statistically significant in any of the models (bivariate and 2-part model, explained later). However, we retained self-rated health despite its nonsignificance in any model because of its strong theoretical significance in the literature for the demand for health insurance [25, 27]. Covariates analyzed in this study included education level (primary/secondary, college, university, and postgraduate), monthly family income (<₮500,000, ₮500,000-₮1,000,000, ₮1,000,000-₮2 500,000, ₮2 500,000-₮4 500,000, and > ₮4 500,000), marital status (single, married, and other, where other is categorized as divorced/widowed/common law), proportion of income spent on medical expenditure (none, < 5%, 5–15%, 15–25, and > 25%), health habits (smoking and self-perceived adequacy of exercise), and satisfaction of social health insurance (yes/no). The question “Are you a current smoker?” had three response options: nonsmoker, past smoker, and current smoker. “Past smokers” were respondents who used to smoke but have quit; respondents who have never smoked were considered “nonsmokers.” Adequate exercise was measured using the question “Do you think you have adequate exercise each week?” (150 min of moderate activity or 75 min of aerobic activity per week was considered adequate exercise). We only used satisfaction of social health insurance in the models instead of all other perceptions on social health insurance due to high collinearity among these variables, and the model evaluation based on Akaike information criterion and Bayesian information criterion suggested no additional model improvement when all 4 variables on perception were added.

### Data quality

We worked with the Mongolian National Chamber of Commerce and Industry, which provided us with a reliable sampling frame. The pretest was performed twice among workers from different fields and professions to ensure that the respondents understood the questions accurately. Within each organization, at least one staff member was assigned by the company to facilitate data collection. Typically, the human resource officers were the staff members assigned to facilitate data collection because they would be more likely to understand the company culture and feasibility of the data collection process. To increase response quality and minimize missing data, we asked the Chief Executive Officers or managers of all organization to allow employees to complete the questionnaire at a particular time. We trained a professional research team from Ach Medical University to screen questionnaire responses before entering the data. Raw data were entered into computers by two independent individuals and were compared to ensure accurate data entry.

### Statistical methods

Given that a WTP being zero would not indicate negative WTP (it is reasonable to assume the lowest value a person’s WTP can take is zero.), we performed a 2-part model with the dependent variable divided into an intensive margin and an extensive margin. The density of WTP denoted as gi(.) is defined as follows:

$$ {\mathrm{g}}_{\mathrm{i}}\left({y}_i|{x}_i\right)=\Big\{{\displaystyle \begin{array}{c}\left\{1-\Pr \left( yi>0\mid {x}_i\right)\right\}\times {f}_0\left(0|{y}_i=0,{x}_i\right)\\ {}\Pr \left({y}_i>0|{x}_i\right)\times {ff}_c+\left({y}_i| yi>0,{x}_i\right)\end{array}}\operatorname{} $$

where *yi* is WTP for an individual I, *f0* is the density of *yi* when *yi* = 0, *fc* is the conditional density of *yi* when *yi* > 0, *x* is a vector of covariates, and *f*_0_(0 ∣ *y*_*i*_ = 0, *x*_*i*_) = 1.

We use logit for the first part and ordinary least squares (OLS) for the second part. Thus,

$$ E\left( yi| xi\right)=\frac{1}{\left(1+{e}^{-{x}_i^{\hbox{'}}\alpha}\right)}\times {x}_i^{\hbox{'}}\beta $$

where *α* denotes the vector for the first-part logit model, and *β* is the vector of parameters for the second-part model. The full marginal effect of the covariates needs to consider both the extensive margin (effect of probability that WTP > 0, ie, willingness to participate in the program) and the intensive margin (effect on the mean of WTP conditional on WTP > 0). The full marginal effect can be solved with the derivative of E (*yi*|*xi*) using the chain rule as follows:

$$ \frac{\mathrm{\partial E}\left({y}_i|{x}_i\right)}{\mathrm{\partial x}}=\Pr \left({y}_i>0|{x}_i\right)\times \frac{\partial E\left({y}_i>0,{x}_i\right)}{\partial x}+\frac{\partial \Pr \left({y}_i>0,{x}_i\right)}{\mathrm{\partial x}}\times E\left({y}_i\left|{y}_i\right\rangle 0,{x}_i\right) $$

We retained interaction terms that were statistically significant in at least any part (logit and OLS) of the 2-part model and dropped the terms that were not significant to ensure that the model was parsimonious. All analyses were performed using STATA statistical software (version 15.0, StataCorp, TX, USA).

## Results

Table 1 provides a comparison of the sample characteristics between genders. Willingness to participate in the first bit and satisfaction with the current social health insurance were not significantly different between genders. Most male and female participants were married. Approximately 12% of women spent > 25% of their income on medical expenditure, and the corresponding percentage was only 6.4% for men.

**Table 1:** Sample characteristics by gender

	Female (*n* = 799)		Male (*n* = 858)		***P*** value
	n %		n %		
Age (Mean, SD)	34.8	9.5	33.1	9.3	< 0.001
Willingness to participate in the first bid					0.677
No	509	63.7	555	64.69	
Yes	290	36.3	303	35.31	
Industry					< 0.001
Mining and quarrying	30	3.75	47	5.48	
Processing industries	198	24.78	223	25.99	
Electricity	59	7.38	113	13.17	
Construction	20	2.5	47	5.48	
Wholesale and retail trade	164	20.53	140	16.32	
Transportation	38	4.76	69	8.04	
Information and communication	61	7.63	74	8.62	
Finance and insurance	35	4.38	34	3.96	
Public administration	77	9.64	55	6.41	
Education services	61	7.63	28	3.26	
Health care	56	7.01	28	3.26	
Total monthly family income					0.013
< 500,000	81	10.14	78	9.09	
500,000-1,000,000	280	35.04	371	43.24	
1,000,000-2,500,000	358	44.81	323	37.65	
2,500,000-4,500,000	64	8.01	69	8.04	
> 4,500,000	16	2	17	1.98	
Satisfied with SHI					0.097
No	553	69.21	561	65.38	
Yes	246	30.79	297	34.62	
Education					< 0.001
Primary / Secondary	160	20.03	261	30.42	
College	67	8.39	95	11.07	
University	458	57.32	427	49.77	
Postgraduate	114	14.27	75	8.74	
Marital status					< 0.001
Single	222	27.78	194	22.61	
Married	487	60.95	608	70.86	
Other	90	11.26	56	6.53	
Proportion of income spent on medical care					< 0.001
none	261	32.67	424	49.42	
< 5%	144	18.02	159	18.53	
5–15%	195	24.41	143	16.67	
15–25%	106	13.27	77	8.97	
> 25%	93	11.64	55	6.41	
Self-rated health					< 0.001
Very good	41	5.13	98	11.42	
Good	394	49.31	468	54.55	
Fair	340	42.55	262	30.54	
Poor	21	2.63	26	3.03	
Very poor	3	0.38	4	0.47	
Smoking status					< 0.001
Nonsmoker (base)	656	82.1	305	35.55	
Past smoker	48	6.01	88	10.26	
Current smoker	95	11.89	465	54.2	
Adequate exercise					< 0.001
No	565	70.71	453	52.8	
Don’t know	106	13.27	127	14.8	
Yes	128	16.02	278	32.4	

### Perception of the current social health insurance

**Fig. 2:**
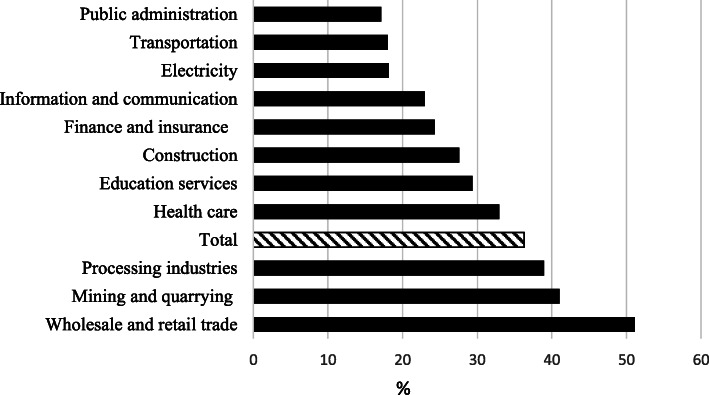
Percentage of the total participants (*n* = 1657) satisfied with the social health insurance stratified by industry

Figure 2 shows the percentage of satisfaction for the current social health insurance stratified by industry. Overall, < 40% of the participants were satisfied with the current system, but when the study population was stratified by industry, a wide range of satisfaction rate was observed, ranging from 17.1% in public administration to 51.1% in the wholesale and retail trade.

Regarding the most desirable aspect of social health insurance, “convenience and ease of access” ranked the highest at 34.27%, followed by “low premium” (29.8%). Only 15.1% of the respondents chose “service quality.” The remaining respondents chose “other” (not specified). Not surprisingly, 40.3% of the respondents selected “low quality” as the most undesirable aspect of the current social health insurance. Regarding the one aspect of social health insurance that the respondent wished to change, 38.1% responded “service,” and 23.22% responded “quality,” representing the top 2 categories of the responses. Table 2 shows perception of social health insurance by whether the subject is willing to participate in the first bid.

**Table 2:** Perceptions on social health insurance by willingness to participate in the first bid

	Total		Willingness to participate in the first bid				*P*-value
			No		Yes		
	n	%	n	%	n	%	
***Are you satisfied with social health insurance? (n = 1657)***							0.003
No	1114	67.23	743	69.83	371	62.56	
Yes	543	32.77	321	30.17	222	37.44	
***Please tick one best thing you like about social health insurance (n = 1608)***							0.196
Premium	479	29.79	305	29.7	174	29.95	
Service quality	243	15.11	158	15.38	85	14.63	
Convenient and easy access	551	34.27	336	32.72	215	37.01	
Other	335	20.83	228	22.2	107	18.42	
***Please tick one worst thing you dislike about social health insurance (n = 1608)***							0.548
Premium	169	10.51	104	10.09	65	11.27	
Service quality	648	40.3	414	40.16	234	40.55	
Convenient and easy access	583	36.26	371	35.98	212	36.74	
Other	208	12.94	142	13.77	66	11.44	
***What would you like to improve in the social health insurance system if you had a chance to do so? (n = 1628)***							0.529
Service	620	38.08	400	38.39	220	37.54	
Quality	378	23.22	231	22.17	147	25.09	
Price	132	8.11	84	8.06	48	8.19	
Health care provider	376	23.1	242	23.22	134	22.87	
Other	122	7.49	85	8.16	37	6.31	

### WTP for parallel private health insurance

Table 3 shows the estimates from the 2-part model. We present the log odds rather than the odds ratio (OR) for the logit part as the model involved interactions, rendering OR difficult to interpret. As expected, higher household income was associated with higher log odds of participation (*P* < .05). Medical expenditure of > 25% of one’s income was associated with higher WTP for participation (₮13,784, *P* < .05). Significant interactions were observed between gender and 3 variables—proportion of income spent on medical expenditure, smoking status, and exercise habits. These effects were more interpretable using marginal effects (Table 4).

**Table 3:** Two-part model for WTP for the supplementary private health insurance

	First part logit			Second part OLS		
	Participate	SE		WTP	SE	
Age	−0.014	0.007	*	193	150	
Sex (male)	−1.125	0.311	***	9697	6383	
Household income	0.214	0.096	*	182	1855	
Sex*household income	0.248	0.132		1101	2538	
Industry						
Mining and quarrying						
Processing industries	0.199	0.278		6992	5897	
Electricity	0.477	0.313		− 620	6538	
Construction	0.909	0.367	*	6542	7281	
Wholesale and retail trade	0.559	0.292		12,768	6036	*
Transportation	0.219	0.333		2543	7025	
Information and communication	0.509	0.314		− 3334	6528	
Finance and insurance	0.966	0.358	**	4586	6743	
Public administration	−0.168	0.334		− 898	7328	
Education services	1.161	0.352	**	2977	6906	
Health care	0.282	0.353		1867	7428	
Satisfied with SHI						
No						
Yes	0.416	0.122		− 1426	2475	
Education						
Primary/ Secondary						
College	−0.125	0.212		6543	4482	
University	0.147	0.149		− 1496	3114	
Postgraduate	0.012	0.222		− 2048	4595	
Marital status						
Never married						
Married	−0.102	0.139		− 347	2776	
Other	−0.390	0.228		1232	4874	
Proportion of income spent on medical care						
none						
< 5%	0.119	0.224		5549	4430	
5–15%	−0.117	0.209		1202	4237	
15–25%	−0.381	0.267		− 3162	5700	
> 25%	−0.164	0.280		13,784	5869	*
Sex* Proportion of income spent on medical care						
< 5%* Male	0.123	0.305		2749	6040	
5–15%* Male	0.743	0.297	*	− 1025	5936	
15–25%* Male	1.324	0.374	***	4576	7545	
> 25%* Male	0.351	0.433		−13,080	9208	
Self-rated health						
Very good						
Good	0.007	0.206		− 1860	4088	
Fair	−0.216	0.225		592	4525	
Poor	−0.499	0.412		2055	8869	
Very poor	0.389	0.816		−13,970	16,288	
Smoking status						
Nonsmoker						
Past smoker	0.488	0.323		8433	5829	
Current smoker	0.153	0.241		265	4846	
Sex# Smoking status						
Male* Past smoker	0.091	0.416		−15,871	7689	*
Male* Current smoker	−0.023	0.291		− 9143	5870	
Adequate exercise						
No						
Don’t know	−0.606	0.249	*	3478	5302	
Yes	−0.143	0.217		4363	4417	
Sex* Adequate exercise						
Male* Don’t know	0.869	0.334	**	− 5121	6944	
Male* Yes	0.494	0.275		− 5481	5546	
Constant term	−1.261	0.423	*	36,982	9141	***

*P ≤ .05; **P ≤ .01; ***P ≤ .001

**Table 4:** Marginal effects

	Total			Female			Male		
	Marginal effect	SE		Marginal effect	SE		Marginal effect	SE	
Age	−66	86		−64	89		−74	88	
Sex (male)	292	1628							
Household income	3482	816	***	2101	1145		4987	1130	***
Industry									
Mining and quarrying (base)									
Processing industries	3749	2743		3892	2830.9		3772	2817	
Electricity	3654	3179		3691	3276		3882	3254	
Construction	10,875	4368	*	11,063	4473	*	11,257	4480	*
Wholesale and retail trade	9597	3089	**	9903	3173	**	9719	3188	**
Transportation	2519	3365		2589	3475		2591	3442	
Information, communication	2894	3161		2878	3259		3167	3236	
Finance and insurance	10,542	4127	*	10,684	4202	*	10,983	4251	*
Public administration	− 1433	3079		− 1469	3181		− 1493	3152	
Education services	11,718	4109	**	11,799	4212	**	12,306	4244	**
Health care	2841	3630		2907	3738		2947	3723	
Satisfied with SHI									
No (base)									
Yes	3429	1502	*	3456	1546	*	3628	1538	*
Education									
Primary/ Secondary (base)									
College	933	2586		1028	2667		824	2630	
University	843	1777		829	1821		923	1811	
Postgraduate	− 598	2567		− 634	2640		− 573	2616	
Marital status									
Never married (base)									
Married	− 1097	1687		− 1115	1727		− 1140	1724	
Other	− 3224	2600		− 3259	2686		− 3398	2650	
Proportion of income spent on medical care									
none									
< 5% (base)	4227	1934	*	3418	2906		4981	2584	
5–15%	2751	1811		− 626	2498		6079	2645	*
15–25%	2975	2331		− 4327	2866		10,004	3651	**
> 25%	2548	2794		3371	3805		1909	3982	
Self-rated health									
very good (base)									
good	− 638	2537		− 672	2605		− 614	2587	
fair	− 1871	2749		− 1891	2830		− 1970	2797	
poor	− 4009	4580		− 4062	4732		− 4225	4660	
very poor	− 2515	9580		− 2795	9816		− 2134	9760	
Smoking status									
Nonsmoker (base)									
Past smoker	5697	2829	*	8618	4627		3047	3386	
Current smoker	−186	1772		1527	2951		− 1788	2016	
Adequate exercise									
No (base)									
Don’t know	− 1105	1850		− 4350	2678		2014	2731	
Yes	1632	1699		285	2737		3109	2152	

*P ≤ .05; **P ≤ .01; ***P ≤ .001

The final effect of each variable on WTP is better interpreted using marginal effects, as both the logit part on likelihood of participation and WTP for participation should be considered. Regarding marginal effects, household income had an overall positive effect on WTP (*P* < .001), and the marginal effect of income for men was higher than that for women. Being satisfied with social health insurance had a higher WTP by approximately ₮1500 (*P* < .05). As expected, a higher proportion of income spent on medical expenditure was associated with a higher WTP for both genders (*P* < .05). However, the marginal effects differed by gender: for men, but not for women, a medical expenditure of 5–25% of income had a positive marginal effect on WTP. Past smokers had a higher WTP by approximately ₮5700 than nonsmokers (*P* < .05). The predicted WTP for men and women was ₮16,369 (SE = 1196, *P* < .001) and ₮16,661 (SE = 1074, *P* < .001), respectively.

### Variation by industry

From the 2-part model, willingness to participate in the first bid and WTP for participation varied by industry. Compared with the mining industry, the participants from the construction, finance, and education industries had significantly higher odds of willingness to participate. The participants from the wholesale industry had a higher WTP than those from the mining industry (*P* < .05).

**Fig. 3:**
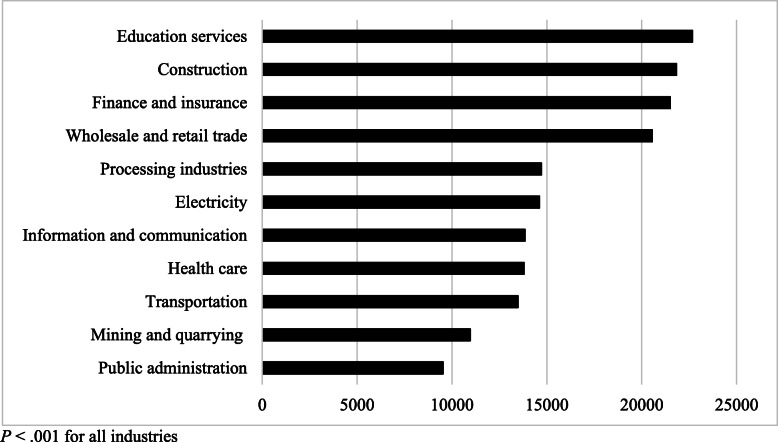
Predicted WTP by industry. *P* < .001 for all industries

Figure 3 shows the ranked predicted WTP given both willingness to participate and WTP for participation (*P* < .001 for all industries). The education industry had the highest predicted WTP ₮22,675 (SE = 3346), and the mining industry and public administration had the lowest.

## Discussion

This study is the first to highlight the possibility of introducing parallel PHI as a complement to the existing SHI system in Mongolia. Mongolia’s healthcare system has rarely been studied. We found that the proportion of workers satisfied with the current social health insurance system was almost equal to that of WTP for the supplementary private health insurance (31% vs. 35%). The predicted WTP for the parallel private health insurance for men and women was Mongolian Tugrik (₮)16,369 (*p* < 0.001) and ₮16,661 (*p* < 0.001), respectively. The highest predicted WTP was found for workers from the education industry (₮22,675, SE = 3346). Income and past or current medical expenditures were significantly associated with WTP.

The public versus private health care financing debate continues in academic and policy circles worldwide [33]. The role of private health insurance differs depending on the country’s wealth and health service development among low- and middle-income countries [34]. Similar hybrid financing systems exist in other countries. For example, in the United Kingdom, approximately 11% of people have had parallel private insurance [35]. Australia and Canada [33] also have similar systems. The results of previous studies in Mongolia have indicated high out-of-pocket expenditure [8, 9], which leads to an immense economic burden on households, leading to poverty [2, 36]. The social health insurance in Mongolia covers mostly inpatient care services, and the coverage is different among public and private health care providers. Services from public health care providers are relatively difficult to access and have inferior quality compared with services from private providers; thus, people are required to pay more for expensive private services. Waiting time is another factor that leads to the use of private health facilities, especially among workers, where time represents the opportunity costs of being sick [37, 38]. Private hospitals that are affiliated with social health insurance still rely heavily on out-of-pocket payments from patients (an average of US$27 per capita on out-of-pocket expenditure) [6, 22]. This amount is most likely unaffordable for an average Mongolian household, given that the average monthly household income was only US$451 in 2018 [22]. The option of financial protection mechanism is very limited and remains a formidable challenge, particularly in developing countries [39].

Empirical results from early studies suggest that demand for health insurance is affected by education [22] and income levels, and the male gender is associated with a higher willingness to pay (WTP) [23, 24]. This prompted us to investigate differences in WTP by gender in Mongolia during the transition period when women had a higher education level [25] but lower wage and fewer job opportunities than men [26]. There are large differences in earnings that cannot be explained by education and experience. The overall gender wage gaps are approximately 10% in all industries [20]. Additionally, early retirement, compared with men, has negative implications for women’s career progression [3]. Moreover, concerning SDG 5 by the United Nations, it is crucial to clarify the current situation on gender equality in Mongolia. Raising awareness of the gender imbalance, especially related to the education level, is essential for policymakers because this topic lies at the intersection of many fields, including education, health, and labor [40], and the position of women is different in different countries [21]. However, the difference between gender in the marginal effect in terms of WTP was not high. This may be because of the current gender imbalances in higher education (14.27% of women had postgraduate education level vs. 8.74% of men, which is also noted in a previous study [40]) and the wage level. By contrast, studies have indicated that men are more willing to pay for health insurance [25].

Income [27, 31], age [27], and medical expenditure burden [27, 31] are essential factors associated with WTP. In our study, the predicted WTP was the lowest in the public administration sector, probably due to the low average income in that sector compared with other industries. Public sector employees may already be enjoying some medical benefits from public health care providers because some government workers have access to specialized medical facilities that provide services only to them on a priority basis. The highest amount stated with the demand of WTP was among workers from education and finance and insurance sectors, which can be attributed to a higher level of education and knowledge of private health insurance [25–27]. We did not find age to be significantly associated with WTP. A previous study reported that WTP decreases with age [25]. This difference may be explained by the fact that the working population in Mongolia is relatively young; hence, the difference between age groups is less salient.

Respondents who were satisfied with the current social health insurance system were more willing to participate than those who were not. The satisfied respondents may have used the social health insurance system before, so they know the benefit of insurance and may plan to increase the service quality and access through the supplementary private health insurance. Health insurance may thus improve the quality of care provided by public health care facilities due to competition with private providers.

This study has some limitations. First, we included only workers from Ulaanbaatar, which may not be generalizable to the entire country. However, 41% of the entire working population lives in the capital city of Ulaanbaatar [22]. Thus, Ulaanbaatar would be the most reasonable place to start such a program in Mongolia. Another limitation can be interviewer bias, as respondents may have equated answering ‘Yes’ to the WTP question as participation and that they are obligated to pay, which can decrease participation in the first bid. This issue, however, is not unique to this study [25].

The Mongolian health care system is underfunded, and its service quality is low, particularly among public health care providers. Improving the health care system is the main priority of the Mongolian government. However, public health sector reform is difficult and time intensive, even in developed countries [41, 42]. In addition, in several Asian countries [43, 44], private health insurance industries are well developed despite efficient public health sectors. Reforming the public health sector however, remins an option.

Policymakers can discuss how to establish a more sustainable health insurance system in Mongolia. We suggest that first, the SHI package should be increased on the basis of the analysis of Mongolian population’s satisfaction, and the quality and access of public health care providers should be improved through competition with private health care providers, and second, public knowledge about the health insurance system should be increased, thus changing the predominantly negative attitude toward health insurance, and allowing private health insurance to complement mandatory social health insurance at the national level through appropriate policy. Because of similarities in the background of the postsocialist countries, our results may be useful for other such countries in the transition period and other developing low- and middle-income countries trying to achieve universal health care coverage. Appropriately managed private health insurance can play a positive role in improving access and equity of health care in developing countries and reducing the risk of poverty due to out-of-pocket health expenditure. In particular, to raise the value of health insurance, policymakers must understand the importance of improving the quality of health care services in Mongolia.

## Conclusion

Our study is the first survey-based study for predicted WTP for PHI for the employed population in Mongolia. The employed population, rather than the general population, can be considered the main contributor of healthcare financing in many developing countries; therefore, information on their WTP for PHI is essential for determining the feasibility of the program. To reduce out-of-pocket health expenditure among the working population in Ulaanbaatar, Mongolia, supplementary parallel health insurance is feasible given that the predicted WTP is around 2.4% of the median salary. However, given high variations among different industries and sectors, different incentives may be required for participation.

## Data Availability

The datasets generated and/or analyzed during the current study are not publicly available due to IRB regulations but are available from the corresponding author on reasonable request.

## Abbreviations

UHC: Universal health coverage
OOP: Out-of-pocket
PHI: Private health insurance
NSO: National Statistics Office
WTP: Willingness to pay
SDG: Sustainable Development Goals
CV: Contingent valuation
OR: Odds ratio
SE: Standard error
SD: Standard deviation
